# Gait Impairment in Traumatic Brain Injury: A Systematic Review

**DOI:** 10.3390/s22041480

**Published:** 2022-02-14

**Authors:** Anthony Dever, Dylan Powell, Lisa Graham, Rachel Mason, Julia Das, Steven J. Marshall, Rodrigo Vitorio, Alan Godfrey, Samuel Stuart

**Affiliations:** 1 Sport, Exercise and Rehabilitation Department, Northumbria University, Newcastle NE1 8ST, UK; tony.dever@northumbria.ac.uk (A.D.); lisa4.graham@northumbria.ac.uk (L.G.); rachel2.mason@northumbria.ac.uk (R.M.); julia.das@northumbria.ac.uk (J.D.); rodrigo.vitorio@northumbria.ac.uk (R.V.); 2 Department of Computer and Information Sciences, Northumbria University, Newcastle NE1 8ST, UK; d.powell@northumbria.ac.uk (D.P.); alan.godfrey@northumbria.ac.uk (A.G.); 3 Department of Tourism, Hospitality, Events and Food, Sheffield Hallam University, Sheffield S1 1WB, UK; steven.marshall@shu.ac.uk; 4 Northumbria Healthcare NHS Foundation Trust, North Tyneside General Hospital, North Shields NE29 8NH, UK

**Keywords:** gait, TBI, concussion, inertial-measurement-unit, wearables, biomechanics

## Abstract

Introduction: Gait impairment occurs across the spectrum of traumatic brain injury (TBI); from mild (mTBI) to moderate (modTBI), to severe (sevTBI). Recent evidence suggests that objective gait assessment may be a surrogate marker for neurological impairment such as TBI. However, the most optimal method of objective gait assessment is still not well understood due to previous reliance on subjective assessment approaches. The purpose of this review was to examine objective assessment of gait impairments across the spectrum of TBI. Methods: PubMed, AMED, OVID and CINAHL databases were searched with a search strategy containing key search terms for TBI and gait. Original research articles reporting gait outcomes in adults with TBI (mTBI, modTBI, sevTBI) were included. Results: 156 citations were identified from the search, of these, 13 studies met the initial criteria and were included into the review. The findings from the reviewed studies suggest that gait is impaired in mTBI, modTBI and sevTBI (in acute and chronic stages), but methodological limitations were evident within all studies. Inertial measurement units were most used to assess gait, with single-task, dual-task and obstacle crossing conditions used. No studies examined gait across the full spectrum of TBI and all studies differed in their gait assessment protocols. Recommendations for future studies are provided. Conclusion: Gait was found to be impaired in TBI within the reviewed studies regardless of severity level (mTBI, modTBI, sevTBI), but methodological limitations of studies (transparency and reproducibility) limit clinical application. Further research is required to establish a standardised gait assessment procedure to fully determine gait impairment across the spectrum of TBI with comprehensive outcomes and consistent protocols.

## 1. Introduction

Traumatic brain injury (TBI) is defined as mild, moderate (modTBI), or severe (sevTBI) injury that results in symptoms that can persist across an acute (days to weeks) or chronic (months to years) time-period [1]. Mild TBI (mTBI), commonly known as concussion, has had predominant focus as it is the most common type of TBI (i.e., mTBI accounts for up to 84% of TBI) [2,3]. TBI can cause deficits in motor and non-motor functions, such as impaired cognitive function, headaches, fatigue, depression, anxiety, and irritability [4]. American Congress of Rehabilitation Medicine [5] describes mTBI as a “mild insult to the head that results in a brief period of unconsciousness followed by impaired cognitive function”. Alternatively, moderate and severe TBI are described as traumatic brain injuries of increased severity lasting a longer period of time [6]. Individuals who present with modTBI express a great variability of injury severity and acute phase injury course, potentially leading into chronic difficulties at a later stage [6]. ModTBI sufferers may exhibit more aggressive symptoms of intra and extracranial injuries with the possibility of inducing secondary brain injury [7]. Furthermore, sevTBI patients demonstrate secondary implications of brain injury such as deviations in physiological variables, namely, systolic blood pressure, oxygen saturation, partial arterial pressure of oxygen, body temperature, serum sodium and glucose [8]. These symptoms can present within both the acute and chronic phases of injury and therefore represent a spectrum of injury. Motor impairments are prevalent, for example, 80% of people who suffer mTBI report balance impairments within days of injury [9], and 30% report chronic (longer term; >12 weeks) symptoms of balance and/or gait impairment [10]. As such clinical assessment of physical and symptom deficits remain an important component of TBI assessment. TBI assessment has traditionally been based on subjective self-reporting or clinical rating of such symptoms, or neuropsychological ‘pen and paper’ testing, and standing balance, tandem gait performance [11]. However, specificity and accuracy of such tests can vary greatly due to the subjective nature in visual assessment and error tracking greatly limiting the replicability and validity of results [12,13,14]. Therefore, recently significant attention has been dedicated to more objective assessment (force plates, and inertial measurement units, IMU) of TBI. Recent evidence has suggested that objective measures of gait may be useful in TBI assessment, as gait has been shown to be a useful biomarker for neurological impairments (e.g., dementia, neurodegenerative diseases) [15,16,17]. Significant barriers limit the objective measure of gait in TBI within clinical practice, as current TBI assessment guidelines recommend the use of subjective/clinical visual assessment [18,19]. Objective gait measurement may be useful for diagnosis and management post-TBI, as it provides sensitive outcome measures for clinical interpretation [20]. However, there are many challenges to the transition of objective gait assessment into clinical settings. Rehabilitation and prognosis is often non-specific to different severities of TBI from mild, to moderate, to severe, which is further complicated by the stage of recovery or since injury of acute or chronic [16]. The spectrum of TBI complicates the use of objective biomarkers, as there needs to be a clear differentiation between sub-groups in order to suggest an outcome is an effective marker of neurological injury. To date, no review has examined the gait impairments in TBI across the spectrum of the condition.

The purpose of this review was to systematically examine the literature on gait impairment amongst adults with TBI across the spectrum of the injury (mTBI, modTBI, and sevTBI). Specifically, this study aimed to examine; (1) how gait was measured; (2) gait outcome measures and equipment used; (3) how does TBI severity impact upon gait metrics. This will help to inform the extent of gait impairment in TBI, as well as whether gait is a useful biomarker and inform clinical assessment/management. Methods of gait assessment will be discussed to determine clinical application.

## 2. Materials and Methods

### 2.1. Search Strategy

**Figure 1:**
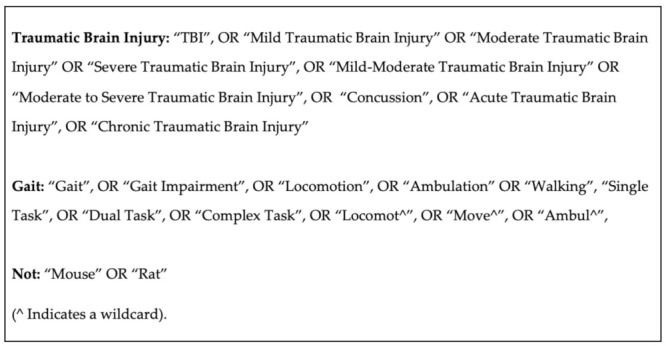
Key Search Terms. Reference to Title, Abstract and Key Terms.

This review follows the Preferred Reporting Items for Systematic Reviews and Meta-Analyses (PRISMA) guidelines. The key search terms were “traumatic brain injury” and “gait”. A catalogue of synonyms was formulated for each key term (Figure 1). Relevant Boolean and medical subject subheadings (MeSH) were applied as seen in Figure 1. The search strategy compromised of four electronic databases: AMED, CINAHL, PubMed and OVID, from 1960 to February 2021. Studies were considered relevant if they incorporated terminology which focussed on gait assessment in TBI and healthy control subjects in the title, abstract or keywords. An initial title screen for relevant articles was performed by the reviewer (AD) once the searched database results had been combined. After initial title screen, both the titles and abstracts of the selected articles were reviewed by two independent reviewers (AD, DP). A review of full text was required if it was not clear from the title or abstract whether the study met the review criteria.

### 2.2. Inclusion and Exclusion Criteria

Articles were included if they reported use of a digital device to measure gait in people with TBI. Studies were included only if they included a control group for comparison to TBI cohorts, so that TBI specific differences could be identified. Articles were excluded if they involved children (<18 years old), participants who had sustained a previous TBI, or a TBI group that did not have any information on the diagnosis (i.e., self-reported history of TBI with no current symptoms), did not provide specific objective gait outcomes from a digital device (i.e., only reported subjective outcomes) and involved a rehabilitation or intervention of some form. Only articles written in English were considered for review and any abstracts, case studies, conference proceedings, reviews, commentaries, discussion papers, or editorials were excluded.

### 2.3. Data Extraction

Data were extracted by the reviewer (AD) then synthesised into table format, with a second reviewer (DP) confirming the data. Data included demographic, instrumentation, study protocol, outcome measures and key findings.

## 3. Results

### 3.1. The Evidence Base

**Figure 2:**
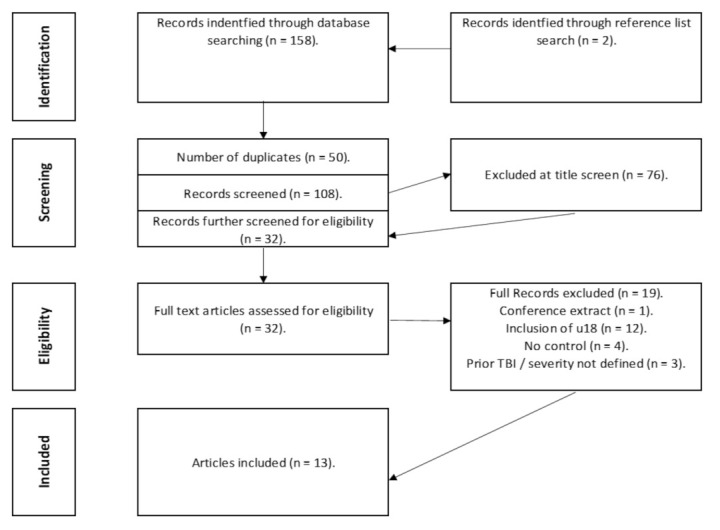
PRISMA flow chart of study search.

The search strategy yielded 156 articles, we excluded 48 duplicates (Figure 2). An initial screen identified 108 articles of interest, but 75 articles were excluded at title screen for not meeting the inclusion criteria and a further 19 were excluded during the full-text screen, with a further five removed at the final review stage. In total, 13 articles were included by consensus from the screening reviewers (AD, DP, and SS). Most of the removed articles were excluded because they included adolescents (Under 18′s), participants who suffered a previous TBI or did not include a healthy control group (full list of excluded articles and reasons located in Supplementary Material S1).

### 3.2. Particpants

The reviewed articles (*n* = 13) investigated individuals who suffered a TBI over acute and chronic time periods across a range of severity from mTBI to sevTBI (Table 1). Most studies (*n* = 6) examined participants with mTBI, with modTBI (*n* = 1) [21] and sevTBI (*n* = 1) [22] less studied. Several studies examined across a range of different TBI severities, specifically; one study investigated modTBI to sevTBI [23], another examined sevTBI and very sevTBI [24], and three studies examined gait in general TBI (combining mTBI, modTBI, sevTBI into one group) [25,26,27]. Five studies examined participants within an acute stage (<7 days), one study was conducted at a sub-acute stage (>7 days) [25] and seven studies examined participants at a chronic stage (>12 weeks). Only one study examined participants across a range of TBI stages from sub-acute to chronic (time since injury ranging from 2 months to 28 months post injury) [23].

In terms of demographic characteristics, the majority of the studies included both males and females, with ages that ranged from 18 to 53 years. One article did not provide specific demographic information for age [25]. There were various inclusion and exclusion criteria for TBI participants (Table 1).

### 3.3. Equipment

Table 2 shows that there was a lack of standardisation in instruments used to assess the characteristics of gait that were assessed in the reviewed studies, with inertial measuring units (IMUs), instrumented gait mats, force plates or motion capture systems all used. Majority of the articles used IMU devices (*n* = 5) to monitor spatiotemporal gait features, which were placed at various locations (i.e., feet, lumbar region, sternum, forehead etc.). The sampling frequencies used to quantify gait performance using IMU’s appears consistent (128 Hz), while motion capture varied between 60 Hz [27] and 120 Hz [22,28,29]. Three studies used force plates at a sampling frequency ranging from 960 to 1080 Hz [21,22,27]. One study used a smartphone to quantify gait speed [30].

### 3.4. Procedures

Table 3 shows that there was a lack of consistency in the specific study protocols, but the majority of the studies included in this review investigated both single and dual-task gait conditions (*n* = 6), while some studies investigated single task (*n* = 4), dual-task (*n* = 1) and complex task (*n* = 2) parameters alone. In terms of dual-task paradigm, eleven articles used a question-and-answer task, including serial subtraction in sevens (*n* = 5), spelling a 5-letter word backwards (*n* = 2), reciting months of the year in reverse order (*n* = 3). Additionally, the audio Stroop test (*n* = 1) and modified Stroop test (*n* = 1) and reading aloud a piece from a newspaper article were used (*n* = 1). Complex gait tasks used obstacle crossing (*n* = 2) with obstacles individualised according to the participants height.

### 3.5. Outcome Measure

There was a lack of standardisation of outcomes reported with reviewed articles providing various outcome measures on spatiotemporal, kinetic, and kinematic markers of gait. The majority of the articles included examined spatiotemporal parameters of gait with the most consistent measures being gait speed (*n* = 9) and measurements surrounding stride (i.e., stride length or stride time) (*n* = 6). Similarly, centre of mass displacement (*n* = 4) was the most common outcome measure used when considering kinematic assessment of gait. Furthermore, regarding kinetic parameters, ground reaction forces (*n* = 3) were reported.

### 3.6. Key Findings

This review identified a variety of methods associated to measuring gait following a TBI (Table 2). For example, when measuring gait using a single task paradigm, this review identified gait speed as a distinguishing factor between TBI participants and controls [22,24,25,31,32]. However, Fino et al. (2016) reported that single-task gait was not different between TBI and controls and suggested that dual-task paradigms are needed to elicit gait deficits [33]. This was seen in studies that examined dual-tasks, as gait impairments were found during dual-task compared to single-task walking across the spectrum of TBI and different acute and chronic stages of the injury. Furthermore, complex gait tasks were examined in several studies and showed that deficits can be found using these protocols. For example, Vallée et al. (2006) determined that TBI participants were slower while performing the Stroop task when avoiding the wide obstacle and walked more slowly for narrow and wide obstacle conditions [23]. Furthermore, McFadyen et al., (2003) also showed an increased lead-limb clearance margins for TBI group throughout all conditions and TBI spectrum [25]. Overall, despite the differences in methodologies between studies, participants with TBI had impairment in gait with single-task, dual-task, and complex task performance in the reviewed studies, which was regardless of severity or stage, but the deficits were selective to particular outcomes within studies and lacked consistency across studies.

**Table 1:** Study populations, time since injury, inclusion/exclusion criteria, and TBI diagnosis.

Author	TBI Population	Controls	Time Since Injury	Inclusion		Exclusion	TBI Diagnosis
Basford et al. [26]	TBI Group: *n* = 10. (F:4, M:6). Age: 40.9 ± 11.3. TBI Severity: Mild: 4/10 Moderate: 2/10. Severe: 4/10.	Control Group. *n* = 10 (F:4, M:6). Age: 41.2 ± 11.4. Matched according to height, age (± 5 years), gender and weight (± 7.5 cm).	Time since Injury: 9 evaluated within 2 years of TBI. 1 had a duration of 15 years and 4 months.	Aged between 18 and 65. Documented TBI injury history and medical records. Decreased GCS Within 24 h of hospital admission with documented loss of consciousness. 3 months post injury. Living as part of the community. Normal gait and balance before injury. Complaints of dizziness or unsteadiness when walking. Review of hospital and radiology records. Normal neurological and musculoskeletal examination.		Cognitive, medical, or behavioural issues.	Mayo Clinic Traumatic Brain Injury Model Systems centre.
Belluscio et al. [24]	TBI Group: (19 RTA’s, 1 Fall) Severe TBI Group: >19 GCS score. *n* = 10 (F:2, M:8) Age: 33.2 ± 9.6. Very Severe TBI Group: ≤ 19 GCS score. *n* = 10. F:3, M:7) Age: 36.1 ± 13.1.	Control Group: *n* = 20. (F:5, M:15) Age: 33.9 ± 9.5.	Time since Injury Severe (days): 308 ± 182. Time Since Injury Very Severe (days): 512 ± 476.	Control group Matched to age, height, and weight. TBI Group. Aged between 15 and 65. Glasgow Coma Scale score ≤ 8. Level of cognitive function ≤ 7. Presence of disturbances in static and dynamic balance. Able to understand verbal commands.		Control Group. Presence of any orthopaedic, neurological or co-morbidities that could influence motor performance.	Physician Glasgow Coma Scale.
Fino et al. [33]	Mild TBI Group: 4 injured. (M:1, F:3) Age: 19 ± 0.8.	Control group. 4 matched control participants. (M:1, F:3) Age: 19.5 ± 1.2.	Time Since Injury (Days): 7.	Recently concussed athletes.		History of mental illness diagnosed cognitive impairment, unresolved acute lower extremity injury. Controls were excluded if they had suffered concussion or brain injury in the last year.	Trained sports medicine physician.
Fino, [29]	Mild TBI Group: (M:2, F:3) Age: 18.8 ± 0.8.	Control group 4 matched control participants. (M:1, F:3) * No eligible control consent gained for 5th participant. Age: 19.5 ± 1.2.	Time Since Injury (Weeks): 6.	Recently concussed athletes.		Unresolved acute lower extremity injury, history of mental illness, diagnosed cognitive impairment.	Medical physician.
Martini et al. [31]	Chronic Mild TBI Group *n* = 65 Age: 39.6 ± 11.7. Time since TBI—1.1 years.	Control Group: *n* = 57. Age: 36.9 ± 12.2 Time since injury: 1.1 years.	Time since injury: 1.1 years	Mild TBI Group. Diagnosis of TBI based on the veteran health affairs/department of defence criteria. Symptoms persisting >3 months. Between 21 and 60 years old.	Control Group Between 21 and 60 years old. No self-reported history of Mild TBI or brain injury.	Any other injury. Medical, substance, neurological illness. Significant hearing loss. Inability to follow direction. Medications that may hamper balance.	Veteran health affairs/department of defence criteria.
McFadyen et al. [25]	TBI Group: *n* = 8 (M:8). Post Traumatic Amnesia (Weeks): 3.9 ± 4.4. Glasgow Coma Scale taken at hospital admission: 8.3 ±4.4. Glasgow Coma Scale Scoring Range: 3 to 14. 1 participant in a coma for 15 days following TBI.	Control Group: *n* = 4 (M:4). Age: Range 22.75 to 44.3. Median: 25.9. No standard deviation or average reported.	Time since injury until time of testing (months): 4.2 ± 1.5.	Recruited from Quebec Rehabilitation Institute. TBI patients capable of walking without aid. Minimum speed of 1 m/s. Control Group. Matched by median age/BMI to TBI participants. No reported physical issues.		Any physiological, musculoskeletal, or neurological disorders other than the diagnosed TBI. Excluded if brainstem or cerebellar damage was present following TBI.	Medical professionals at Quebec Rehabilitation Institute.
Oldham et al. [30]	Mild TBI Group: *n* = 50 (F:32, M: 18). Age: 20.2 ± 1.27.	Control Group: *n* = 25. (F: 13, M:12) 21.1 ± 2.2.	Time since injury: 72 h.	Concussion Group. Active member of NCAA team. Medically cleared for participation before pre-season testing.		Neurological disorder. Current/previous lower extremity injury. Vision disorder. Previous concussion in last 6 months.	Certified athletic trainer confirmed by a team physician. In check with the 5th international conference on concussion in sport.
Parker et al. [21]	University/college athletes, club sport athletes. Moderate TBI Group: *n* = 29 (Suffered grade 2 TBI according to the Academy of Neurology Practice). Age: 21.6 ± 3.26. (F:14, M:15)	Control Group: *n* = 29 Age: 21.38 ± 3.38. (F:14, M:15).	Time since injury to initial testing (hours): 34.26 ± 11.78.	Suffered grade 2 (moderate) concussion. Identified by certified athletic trainers. Control group matched by height, age, gender, and physical activity.		History of neurological diseases. Uncorrected visual impairment. Persistent vertigo symptoms. Experienced consistent unsteadiness, light-headedness or falling.	Certified Athletic Trainers, USA.
Parrington et al. [32]	53 Participants (Collegiate Athletes across 6 sporting departments in various universities). Mild TBI Group: *n* = 23 *n* = 2 did not return to play during specified 8-week period. (F:5, M:18) Age: 20.1 ± 1.3. Contact: Non-Contact Sport. 18:5.	Control Group: *n* = 25 (F:6, M:19) Age: 39.3 ± 13.0. Contact: Non-Contact Sport. 12:13.	Time since injury: 24–48 h. Time to return to play (days): 13.7 ± 4.4.	18 years or older. Received a diagnosis from mentioned medical physicians. Diagnosed using the Sports Concussion Assessment tool. Control Group: Student athletes competing in same university and departments. Matched to height, age, gender, and mass where possible.		Medical condition that would impair cognitive ability or mobility. Injury within the 6 months prior to study commencing. Surgery within 6 months prior to the beginning of the study.	Team clinician. Oregon Health and Science University sports physician.
Pitt et al. [34]	Mild TBI Group: *n* = 11 (F:7, M:4). Age: 20.1 ± 1.3	Control Group: Healthy matched—*n* = 11 (F:7, M:4). Age: 20.6 ± 1.9.	Time since injury: 72 h.	Concussed participants matched to healthy control by sex, age, height, and weight.		Injury affecting normal gait. History of permanent memory loss. Concentration abnormalities. Impaired hearing. Potential controls who sustained an injury in the last year.	Physician at university health clinic.
Shan Chou et al. [27]	TBI Group: *n* = 10. (F:4, M:6). Age: 40.9 ± 11.3. 4 suffered mild TBI (GCS > 12). 2 suffered moderate TBI (GCS = 9–12) 4 participants suffered severe TBI (GCS < 9).	Control Group: *n* = 10 (F:4, M:6). Age: 41.2 ± 11.4. Matched with age, gender, height, and weight.	Time since Injury: 9 evaluated within 2 years of TBI. 1 had a duration of 15 years and 4 months.	Individuals who suffered a TBI.		Abnormal neurological and musculoskeletal examinations. Cognitive problems. Medical problems. Behavioural problems.	Based on medical records and history. Glasgow Coma Scale (GCS).
Vallée et al. [23]	18 Participants: Moderate to Severe TBI Group: *n*= 9(F:1, M:8) Age: 39.3 ± 13.0.	Control Group: *n* = 9 (F:1, M:8) Age: 39.7 ± 12.3.	Time since injury (Months): 8.6 ± 5.7.	Only 1 TBI. Severity ratings of moderate to severe based on GCS score, duration of posttraumatic amnesia, length of loss of consciousness, examination neuro diagnostic examination. Able to walk at a speed >0.7 m/s without aid. Control participants—no self—reported physical or neurological issues.		Skull fracture/Cerebral Lesion caused by a perforation. Cognitive behavioural issues. Behavioural issues. Neurological or Musculoskeletal issues that affect locomotion.	TBI Unit of Quebec Rehabilitation Institute.
Williams et al. [22]	TBI Group: *n* = 41 (F:10, M:31) Age: 29.1 ± 9.4 Time since injury (days): 2609.4 ± 2327.3. Posttraumatic Amnesia (days): 84.9 ± 57.5. HiMAT Score: 22.7 ± 11.5.	Control Group: *n* = 25. (F:9, M:16) Age: 27.8 ± 7.4.	Time since injury (days): 2609.4 ± 2327.3.	Sustained a TBI. Able to walk independently over 20 m without the use of a gait aid.		Unwilling or unable to provide informed consent. Concurrent central nervous system disorders. Severe cognitive or behavioural problems that prevented assessment.	Medical Facility (Hospital).

**Table 2:** Study aims, procedures, equipment, outcomes, and findings.

Author	Aims	Procedures	Equipment	Outcome Measures	Key Findings
Basford et al. [26]	Assess the gait and dynamic balance of individuals with instability or imbalance after TBI. Examine the relationship between symptoms.	Tinetti Balance Assessment Dizziness Handicap Inventory. Dix–Hallpike Test. Caloric Irrigation. Optokinetic Testing. Pure-Tone Hearing Testing. Computerised Dynamic Posturography. Motion Analysis/Single Task Gait. Attached to safety harness. Barefoot walking along 10 m walkway at self-selected pace. 27 reflective markers placed on bony landmarks. 3 trials by each participant. Data analysed from heel strike to heel strike of same limb. Biomechanical Model. 13 body segments. 4 upper extremities. 6 lower extremities. 1 pelvic, trunk, and head.	8—Camera ExpertVision system. 60 Hz sampling rate.	Dizziness Handicap Inventory (DHI), Caloric irrigation, Optokinetic testing, Dix–Hallpike Test, Posturography, Centre of mass (COM) movement	Significant differences in gait parameters noted between participants with TBI and without. TBI sufferers exhibited lower anterior, posterior, higher medial, and lateral centre of mass displacement and velocity.
Belluscio et al. [24]	Quantify gait patterns in severe traumatic brain injury through wearable inertial sensors. Investigate the association of sensor-based quality of gait indices with the scores of administered scales.	Clinical Assessment: Dynamic Gait Index. Berg Balance Scale. Community Balance and Mobility Scale. Motor Assessment. 10 m Walk Test. Figure of 8 Walk Test. Fukuda Stepping Test. Inertial measurement location Occipital cranium bone. Centre of sternum. L4/L5 Level of spine. Bilaterally on shanks above lateral malleoli.	5 inertial measurement units. (Opal, APDM, Portland, Oregon, USA). 128 Hz.	Berg Balance Scale. Community Balance and Mobility Scale. Spatiotemporal: Dynamic Stability. Symmetry. Smoothness. Temporal: Stride Frequency. Stride Duration. Fukuda Step Test: Rotation (Degrees). Side Rotation (% Right). Anterior/Posterior Displacement. Medio-lateral Displacement.	Significant differences exhibited in the three motor tasks between control group and both severe groups and between severe and very severe group. Statistically significant differences seen between control group and severe TBI were found in spatiotemporal parameters of Fukuda Step Test. No differences noted in terms of lateral/forward displacements among the three groups. Or among amount of rotation or side rotation among the three groups. Significant differences in walking speed noted between control group and both severe groups and between severe and very severe group.
Fino et al. [33]	To determine the local dynamic stability of athletes who recently suffered a TBI during single and dual-task gait.	Weekly tests for 6 weeks at 7 ± 0, 16 ± 1, 23 ± 2, 29 ± 1, 36 ± 2, and 45 ± 3 days following TBI. One year follow up 363 ± 42 days. Sessions occurred in a gymnasium on clean hardwood flooring. Barefoot 18 m straight segment with a pylon at the beginning and end of each section. 14 laps 14 bouts of straight single task gait. Dual-task: Randomly assigned a number between 900 and asked to repeat the procedure subtracting in sevens. 14 bouts of dual-task gait.	Two six-axis inertial measurement units fitted and aggregated in the Technology Enabled Medical Precision Observation (TEMPO) platform. 128 Hz. Placed over xiphoid process and forehead.	Steps identified using trunk vertical accelerations. Stride length—Identified every two steps. Gait Speed—Average time per condition to complete 18 m walk.	Dual-task gait impaired following TBI. No difference between groups during single task gait. No difference in stride time variability. Addition of cognitive dual-task influenced stability in TBI group. TBI group displayed larger local dynamic stability dual-task costs post-TBI. TBI athletes walked slower than controls. TBI athletes increased speed over time (resolved at 1 year follow up)
Fino, [29]	To determine single and dual-task turning kinematics in TBI and healthy athletes.	Weekly tests for 6 weeks at 7 ± 0, 16 ± 1, 23 ± 2, 29 ± 1, 36 ± 2, and 45 ± 3 days following TBI. One year follow up 363 ± 42 days. Barefoot on a wooden floor. Single Task: 18 m × 3.5 m course consisting of several pre-planned 90° turns. 7 laps in each direction around the course. Dual-task Serially subtracted in sevens from a random number between 999 and 900. 14 turns (7 Left and 7 Right). Step and Spin turns clearly defined. 30 consecutive missing frames were discarded.	Four motion capture cameras (ProReflex MCU 170 120, Qualisys Medical AB, Gothenburg, Sweden). Reflective Markers placed on xiphoid process, calcaneus and T9 vertebra. 120 Hz filtered using phase-less fourth-order Butterworth filter—6 Hz cut off.	Stride characteristics Stride Width Stride Length Stride Time Body Orientation Mediolateral Inclination angle at first, second and third heel contact of turning stride.	Path Trajectory: Decreased velocity gain factor in TBI injured athletes relative to controls. Stride Characteristics: Locomotor dual-task cost in TBI group increased stride width, time, widening and slowing of stride during dual-task. Body Orientation: TBI athletes increased their inclination towards the turnover time. TBI injured participants displayed less medial inclination towards step turns and less lateral inclination towards spin turns.
Martini et al. [31]	Determine if gait domains are different without and with chronic Mild TBI. Determine if adding dual-task exacerbates differences in gait across the domains. Determine if self-reported severity scores are related to gait performance.	Single and dual-task conditions: Walk at a self-selected comfortable pace. Each walk was 8 laps × 13 m walk-way with 180° turns. Audio Stroop Test: Headphones in situ, participants listened to an audio stimulus consisting of the words high and low. Randomly paired incongruently or congruently with pitch of voice. Stimulus was delivered every 2.25 s. Symptom Assessment: Neurobehavioral Symptom Inventory (NSI).	Inertial Sensors. Inertial sensors (Opal Sensor, APDM Inc., Portland, OR, USA); Placed on each foot, forehead, lumbar vertebrae and over sternum.	Single Task and Dual-task. Pace. Variability. Rhythm. Turning. Dual-task Cost: Turning. Pace.	Individuals with chronic Mild TBI exhibit deficits across a multitude of gait characteristics. Slower pace and turning at both single and dual-task gait. Less rhythm under dual-task gait conditions. Severe symptoms such as increased gait variability, decreased pace and turning are indicative in chronic TBI group. NSI significantly linked to gait variability in single and dual-task gait.
McFadyen et al. [25]	To definitively understand residual locomotor effects following a TBI on obstructed and unobstructed walking.	Locomotor Capacity and Gait: Gait speed calculated by time to complete 10 m. Dynamic Gait Index. Glenrose Ambulation Index. Balance: Berg balance scale. Time single legged stance. Performed twice on each side with eyes open and closed. Triads of noncolinear infrared markers attached to legs, feet, pelvis, trunk, and head. Participants walked along a 9 m walkway at self-selected pace unobstructed followed by obstructed. Obstruction: Obstacle placed in the middle of the walkway at a moderate height. 122 cm wide × 2 cm deep. Height adjusted to approximately 15% of participants lower limb length. Minimum 5 trials per condition undertaken. Lead and trail limb clearly defined.	Optotrak system (model 3020; NDI Inc, Waterloo, Ontario).	General Cadence—Steps per minute. Gait Speed—Stride length divided by stride time. Bilateral Stride Lengths—Consecutive heel contacts. Toe Clearance during obstructed (Distance above the obstacle normalised to height) and unobstructed (absolute distance above the floor). Maximum joint angle during swing phase. Walking toe and heel proximities—Distances from the obstacle immediately before and following clearance and normalised to stride length.	TBI sufferers walked slower than healthy controls. Greater foot clearance over obstacle noted in TBI sufferers across all conditions. Slower walking was due to decreased stride length and not cadence. Higher foot clearance due to trail foot placement being further from the obstacle and increased hip flexion angles during avoidance.
Oldham et al. [30]	Examine whether changes between baseline and acute post-TBI single task and dual-task tandem gait performance differed between male and female athletes.	Tandem gait measures recorded consistently with SCAT-3. Single Task: Walk heel to toe along a 3 m long line following verbal cueing as quickly as possible. Complete 180° turn and return to start point. Dual-task: As above. Spelling 5 letter words backwards. Subtracting in 6 s and 7 s from a 2-digit number. Listing months in reverse order. Concussed athletes completed trial 1 at preseason testing. Trial 2 72 h post-concussion. Control group completes trial 1 at preseason testing. Trial 2 72 h post trial 1.	Time recorded using smartphone. NR of inertial sensors.	Gait Speed. Single Task time to completion (seconds) Dual-task time completion (seconds). Cognitive accuracy (%).	There were no significant differences for ST or DT tandem gait performance from Time 1 to Time 2 between male and female athletes. Gender was not a determinant of time to completion in collegiate athletes or healthy population. Significant differences between females and males on the amount of change between pre- and post-injury assessments. TBI group demonstrated greater tandem gait impairments (i.e., a positive change in time) between Time 1 and Time 2 than the healthy controls.
Parker et al. [21]	Examine the relationship between measures of dynamic motor performance (single and dual-task walking) and neuropsychological function following concussion over the course of 28 days.	Gait Stability Testing. All TBI athletes were tested 48 h, 5 days, 14 days and 28 days post injury. Control Group tested at the same time points of the study. All participants were tested barefoot and walked on a 10 m walkway at a preferred walk speed. Gait Protocol: Remained the same for each testing day. 10 m level walking under single and dual-task conditions. Single Task: Walk on walkway undistracted with no cognitive requirements. Dual-task: Walk on walkway undistracted while completing a cognitive task. Spelling a 5-letter word backwards. Subtraction by sevens from a random number. Reciting months of the year in reverse order. Each cognitive was completed by each participant and rotated over the testing period. Assessing Gait Variables: 31 reflective markers were placed on bony landmarks. Whole body COM position was calculated as the weighted sum of each body segment (head-neck, trunk, pelvis, arms, forearms, thighs, and feet). Velocity of the COM estimated through cross-validated spline algorithm. COP was calculated, ground reaction forces were collected. Neuropsychological Testing: Assessed at the same time intervals as gait testing with the Immediate Post-concussion Assessment and Cognitive Testing battery (ImPACT; ImPACT Applications, Pittsburg, PA, USA).	8 Camera 3D motion capture system (Motion Analysis Corp., Santa Rosa, CA, USA). Visual markers estimated using—EVaRT 4.37A (MotionAnalysis, Santa Rosa, CA) 4 s at 60 Hz. 2 force plates—(Advanced Mechanical Technology, Watertown, MA, USA). 960 Hz for 4 s.	Neuropsychological testing Processing Speed. Visual Memory. Symptom Score Choice Reaction Time: Average speed of responding to Symbol-matching, Colour-matching and Left–right side matching tasks comprised the score. Variables were examined in one gait cycle, which was defined as heel strike on the force plate to the next heel strike of the same. Four gait stability variables utilised for comparison with NP measures: COM displacement Peak velocity in the medial-lateral direction (MLdisp; MLvel), average gait velocity (GV), the maximum separation between the COM and COP in the anterior direction.	TBI group had significantly greater sway for the dual-task condition on days 5 and 28. The dual-task condition produced significantly faster sway than the single-task condition for both groups, even at 28 days following initial testing Maximum anterior COM–COP separation distance revealed a task effect with the dual-task producing a smaller separation distance than the single-task for the TBI group on all days Visual memory—TBI group showed significant improvement from day 2 to 5 and from day 5 to 14. Group differences were detected for the testing days 2 and 5 with the TBI group performing worse than controls. The TBI group mean processing speed was significantly faster on day 5 compared with day 2 but did not change significantly after day 5
Parrington et al. [32]	Evaluate the recovery of gait and balance in concussed athletes to account for changes in trends following return to play.	Inertial sensors attached bilaterally on anterior and distal aspect of each shank and posterior pelvis at L5. Participants were assessed during 9 testing periods over the course of an 8-week period. 2 testing session in week 1 followed by weekly testing for the next 7. To maintain consistency across testing sites, sessions were performed in well-lit straight hallways on a firm surface. Each session included instrumented balance and gait assessment. Balance: Balance Error Scoring System. Sway Metrics. Gait: Instrumented 2-min walk under single-task and dual-task conditions. Walking normally participants were instructed to walk at a self-selected pace along a 25 m hallway. Single Task: Walk 25 m at self-selected pace. Dual-task: Walk 25 m at self-selected pace while reading aloud a piece from a newspaper article. Reading Task: Completed firstly completed a timed trial in seated position, then during dual-task conditions. Dual-task cost was calculated using words read during baseline and dual-task conditions.	Inertial Sensors—3 wireless Opal; APDM Inc, Portland, OR at 128 Hz. Mobility Lab Software (version 1; APDM Inc34). Trials were video recorded using a (Bloggie Touch; Sony Corporation, Tokyo, Japan) camera. Newspaper articles -Flesch reading level of 72.4 and 78.2 and printed on A4 sheets with font 12.	Balance Error Scoring System (BESS). Sway. Single Task Gait Speed. Dual-task Gait Speed (Walking while reading a handheld article). Dual-task cost of reading on gait speed. Dual-task cost of walking on reading.	BESS: No significant interactions between groups. Sway: Initial differences were observed with TBI group swaying more than control participants. Single Task Condition: Speed did not differ between groups. Gait speed over time was more pronounced in TBI participants. Gait speed stopped increasing at RTP time point in both groups with greater change being seen in TBI group. Dual-task Condition: No initial differences between groups for dual-task speed. Overall gait speed was increased with a more prominent increase in TBI group. After RTP gait speed stopped increasing in both groups.
Pitt et al. [34]	Provide an objective description of angular velocity and acceleration profiles along orthogonal axes from one IMU situated on L5 vertebrae. Demonstrate that detectable differences could be identified in IMU metrics and be utilised to distinguish individuals with a TBI during dual-task walking.	TBI participants: Completed a post-TBI symptom survey (PCSS). Dual gait balance control assessment at five post injury time points—72 h, 1 week, 2 weeks, 1 month, 2 months post-TBI. Dual-task condition: 7 m walk at self-selected pace. Protocol was automated using Superlab 5 Software. Verbal commands and auditory Stroop task delivered through single earpiece Bluetooth device. Stroop Task: Four auditory stimuli high and low spoken in high or low pitch Congruently or incongruently. Participant responded to the audio stimulus. One single stimulus was manually triggered on every third heel strike. Sensors were attached at lateral ankles and over L5 vertebrae. Gait cycles were recorded and processed with the 3rd, 4th, and 5th heel strike.	Superlab 5 software: Cedrus Corp, San Pedro, CA, USA). Blue tooth wireless headset: (Blue Tiger USA, TX, USA). Motion Analysis: OPAL Motion analysis, motion studio software. (APDM, Inc, Portland, OR, USA). IMU data sampled at 128 Hz and streamed to wireless hub. Zero lag, low pass Butterworth filter with 12 Hz cut off.	Peak velocities Medial Lateral Direction: Anterior Posterior Direction: Vertical Direction: Angular velocity around the vertical axis. Angular velocity around the anterior posterior axis. Angular velocities about the vertical axis.	Healthy and TBI participants were distinguished across the two-month post-TBI period through. Angular velocity about the vertical axis. Angular velocity about the AP axis. Peak angular velocities at heel strike. Peak angular velocities during early single leg support distinguished TBI from healthy participants across the 2-month period.
Shan Chou et al. [27]	Determine the possibility of quantitatively assessing dynamic stability that did not have an obvious neuromuscular origin in individuals who suffered a TBI.	Unobstructed level walking. Performed barefoot and a 6 m walkway. Obstacle Crossing: Obstacles set at 2.5%, 5%, 10%, and 15% of each individualised height. Participants were allowed to lead over obstacles with preferred leg.	Reflective markers set at 27 bony landmarks. Eight camera ExpertVision system (Motion Analysis Corp, Santa Rosa, CA). 3D marker trajectory data collected at 60 Hz. Low pass filter using fourth order Butterworth filter—cut off frequency 8 Hz. Ground reaction forces. Two force plates—(Kistler 9281B and Bertec 4060A). 960 Hz.	Gait Velocity. Stride Length. Step Width. Centre of Mass Displacement	TBI suffers walked with significantly lower gait speed and presented with a shorter stride length in comparison to matched controls. TBI elicits greater and faster medio-lateral centre of mass motion and significantly maintained medio-lateral separation distance between centre of mass and centre of pressure when compared to their matched controls.
Vallée et al. [23]	Establish the effects of increasingly demanding environments related to simultaneous visual tasks and physical obstructions to locomotor ability of people who have suffered TBI.	Visual Acuity: Snellen Test. Lab Session: 3 Physical Conditions with 3 concurrent visual tasks. The simultaneous visual stimuli: Adapted versions of the Stroop bar and word tests. Head movement controlled when walking by: 2 columns Coloured bars or words that was displayed simultaneously on the computer monitors placed along the walkway. Participants sequentially state the colour of the 8 bars shown. To increase complexity words were presented that indicated the colour but in a different colour to lexical meaning. Participants were asked to ignore the meaning and state the colour of ink. Physical Condition: 11 m walkway stepping over a narrow obstacle and over a wide obstacle. Obstacle dimensions set to ratio of participants maximum step height and length (Individualised difficulty). Calculated over 2/3 steps with depth and height of obstacles set to 30% of the respective data. 3 trials were performed. Stroop when seated. Participants familiarised themselves with walkway × 2/3 trials. Participants were exposed to 5 trials of each physical condition (unobstructed, narrow, and wide obstacles). 10 trials of each physical condition with visual stimuli randomly presented.	Kinematic Data. 3 Optotrak sensor bars. Frequency: 75 Hz. Statically digitised the heel and toe in relation to foot markers. Microphone. Earphones. 5 flat screen monitors (43.2 cm). Recording computer 1000 Hz.	Reading Times for Stroop bar and Stroop word tasks. Walking Speeds. Stride Length. Obstacle Clearance Margins.	TBI Group slower in performing Stroop bar task during sitting. TBI Group slower while avoiding narrow obstacle. TBI Group slower while performing Stroop task while avoiding wide obstacle. TBI sufferers walked more slowly for narrow and wide obstacle conditions alongside dual-task of highest complexity. Increased lead-limb clearance margins observed for TBI group throughout all conditions.
Williams et al. [22]	Identify the most common gait abnormalities following a TBI and determine their rate of incidence.	25 reflective Pelvis and lower limb. 3 markers placed overlying T2, T10 and sternal notch. Used to define joint centre location. Participants performed walked over a 12 m walkway at a self-selected pace. Spatiotemporal, kinematic and kinetic data across 5 trials were recorded. Speeds effect on kinetic and kinematic data controlled through controls walking at ±5% of TBI self-selected walking speed. Only trials within the 5% were included for analysis. Clinical measurement for mobility—HiMAT.	Kinematic: Motion Analysis: 25 small reflective markers 3DGA. Vicon 512. 8 Cameras. Sampling at 120 Hz. Ground Reaction Force. 3 AMTI force plates. Sampling rate 1080 Hz.	Spatiotemporal. Velocity Cadence. Step Length. Step Duration. Double Support. Base of Support. Kinematic. Trunk Flexion. Trunk Lateral Flexion. Anterior Pelvic Tilt. Pelvic Obliquity. Pelvic Rotation. Hip Extension. Hip Adduction. Knee flexion at initial contact. Knee Flexion Mid-stance. Knee Flexion Swing. Ankle Flexion at initial contact. Foot Equinovarus. Centre of Mass Displacement. Kinetic. Push off Terminal Stance.	Individuals with TBI demonstrated significantly slower walking speed. Additionally, TBI sufferers demonstrated differences in cadence, step length, stance time on affected leg, double support phase, width of base of support. Biomechanically abnormalities were noted with TBI suffers exhibiting excessive knee flexion at initial foot contact. Significantly increased trunk anterior/posterior amplitude of movement, increased anterior pelvic tilt, increased peak pelvic obliquity, reduced peak knee flexion at toe-off, and increased lateral centre of mass displacement were seen in TBI suffers.

**Table 3:** Objective gait task paradigm.

Article	Single Task	Dual-Task	Complex Task
Basford et al. [26]	✓		
Belluscio et al. [24]	✓		
Fino et al. [33]	✓	✓	
Fino, [29]	✓	✓	
Martini et al. [31]	✓	✓	
McFadyen et al. [25]	✓		
Oldham et al. [30]	✓	✓	
Parker et al. [21]	✓	✓	
Parrington et al. [32]	✓	✓	
Pitt et al. [34]		✓	
Shan Chou et al. [27]			✓—obstacle crossing
Vallée et al. [23]			✓—obstacle crossing
Williams et al. [22]	✓		

A notable methodological limitation was found when considering gait impairment across the spectrum of TBI. Specifically, none of the reviewed studies examined gait deficits in TBI across the full spectrum of the injury (mTBI to sevTBI), with several studies combining TBI severities into a single TBI category rather than defining and assessing specific sub-groups. Therefore, there was no evidence on how gait differs between different severity levels of TBI, or if there are consistent deficits that become worse with increased TBI severity.

## 4. Discussion

To the authors knowledge, this review represents the first systematic synthesis of the literature examining gait impairment across the spectrum of TBI. Here we examined 13 studies that reported gait assessments in healthy controls and TBI participants specifically (i) how gait was measured; (ii) gait outcome measures and equipment used; (iii) how does TBI severity impact upon gait metrics.

### 4.1. Instrumentation

There was a lack of standardisation of instruments used to examine gait, therefore gait performance was quantified using several different technologies, but largely motion capture systems or IMUs were favoured over instrumented gait mats or force plates. Motion capture systems are a traditional approach to gait assessment, which are expensive, time consuming to set up, require specialist training and are often limited to specialist research centres or supervised laboratory surroundings, which may not be scalable to low resource settings [35]. Therefore, findings and conclusions drawn cannot be applied, relate or be replicated in other real-life contexts. Alternatively, IMUs have been suggested to overcome this challenge as they are easily implemented, low cost and portable [36], with excellent validity and reliability for gait assessment [37]. Progression to use of IMUs was seen in the majority of reviewed articles, as most used IMU’s to measure gait in TBI [24,30,31,32,33,34,38] which were reported to be a viable and reliable method of gait assessment. IMU’s (and 3D motion capture) were shown to detect abnormalities in gait and provide an overall account as to an individual’s gait cycle following a TBI, as all of the reviewed studies showed gait differences between those with TBI and healthy controls.

### 4.2. Outcome Measures

Gait can be characterised into spatiotemporal, kinematic, or kinetic outcome measures that are underpinned by selective neurological mechanisms [39]. There was a lack of consensus on the approaches used in assessing and reporting gait impairment in TBI, but studies generally reported spatiotemporal and kinematic outcomes. There were a wide range of gait outcomes reported between studies, but most studies reported on a limited amount of selected gait characteristics. The lack of standard assessment and reporting limits the generalisability of the findings, and does not support the use of quantitative methods of review reporting (i.e., meta-analysis) due to risk of bias. The most consistently reported outcome was gait speed (or velocity/pace). Gait speed is a measure of global walking performance [30,40], and is essentially an accumulation of multiple gait features that cannot be accurately quantified with a single outcome measure (e.g., speed [41]). As a result, gait speed in isolation is not a disease specific outcome and it does not reflect the subtle and precise underlying neural mechanisms involved in gait, which requires a more comprehensive examination of multiple gait outcomes. Despite gait speed being used in several studies we are unable to definitively report that it is useful at differentiating TBI groups, as the differences in methodologies (i.e., instruments, protocols, outcomes etc.) mean that we cannot directly compare outcomes across studies, and future work is needed to standardise procedures.

Different underlying brain regions control different aspects of gait [42,43] and therefore with TBI of different regions and severity there is a need for comprehensive gait outcome measure assessment and reporting. Gait is underpinned by a complex system of neural cortical and sub-cortical networks [44] and impairment of any of the specific elements of the networks involved can result in impairment. Comprehensive reporting of gait in TBI literature is limited by the cohort sizes that have been examined, as there are many outcome measures that can be assessed and reported, but small samples sizes limit reporting capabilities and may lead to statistical errors. Many of the reviewed articles in this review had small TBI cohorts (*n* < 20) and as a result the number of outcomes reported may have led to inappropriate statistical analysis or reporting, due to the number of statistical comparisons [45]. There have been attempts to control for the number of comparisons made by using gait models within TBI cohorts (i.e., statistical analysis to reduce data in order to avoid statistical error issues [15]). However, only one of the reviewed articles [31] used a data reduction technique to assess gait outcomes, which highlights the emerging nature of gait assessment and reporting in this field. The development of an outcome measure framework would enable a hypothesis-driven research plan aiming to explain gait disturbance and examine the effect TBI on gait performance across the spectrum. Thus, leading to a greater consensus on most sensitive and accurate gait measure within TBI.

### 4.3. Protocols

There was a lack of consistency when reporting basic methodological procedures in classifying TBI severity, time scale (acute, sub-acute and chronic) and inclusion and exclusion criteria, which limits the generalisability and understanding of results. However, findings suggest that gait is impaired in TBI across the spectrum from mild (concussion) to severe injury status. Despite gait impairments being found, there was a lack of standardisation of procedures that limit the future implementation of gait assessment protocols.

All articles included in this review (*n* = 13) were undertaken in a gait laboratory setting. While laboratory assessments allow for complete experimental control that may uncover gait deficits, the environment may lack functional validity as it may not reflect ‘real-world’ gait [46]. Specifically, assessment of gait in a laboratory setting may fail to capture subtle deficits due to TBI that may ensue within usual environments (e.g., home, community, clinic, work, sports pitch/field, etc.), where there are multiple distractions and a vast array of environmental information to process to complete tasks effectively and safely [21,47]. None of the reviewed studies made the progression to examine gait outside of the laboratory within free-living environments, which has been conducted with physical activity and turning characteristics in previous TBI studies [48], which limits the understanding of the functional impact of potential gait impairments following a TBI.

There is no ‘gold-standard’ protocol for assessment of gait in TBI, as studies used a variety of tasks in an attempt to uncover deficits (e.g., single-task, dual-task, complex tasks etc.). The variety of experimental protocols employed across the included tasks of various complexity that sought to uncover specific TBI-related deficits. For example, single-task gait along a straight path was used in the majority of studies as this is thought to be a ‘baseline’ task that is controlled subcortical processing with minimal executive control [46], which can then be used to compare with more complex gait tasks that may elicit subtle deficits following a TBI. Dual-task gait was commonly used as a more difficult gait task that requires simultaneous cognitive and motor processing involving the executive function [49], which was compared to single task gait and healthy control gait to uncover TBI deficits. The least common gait assessment protocol was complex gait tasks, such as obstacle crossing, which require higher order cortical planning in order to plan and execute obstacle avoidance during walking [50]. The lack of a standardised protocol limits the generalisability of results across studies (i.e., even single-task walking was conducted for different times and distances), which means that quantitative analysis of the outcomes across studies is inappropriate until a common protocol is developed.

Gait provides a simple marker for an individual’s overall health and is a widely accepted predictor of quality of life, decline of cognitive proficiency and falls [51]. Due to neurological decline and difficulties with age, gait becomes a more difficult task to perform efficiently and economically causing a transfer from an automatic to a cognitive level of control in order to execute and perform within a complex environment [39,52]. Increased task complexity for gait assessment was thought to increase the sensitivity of gait analysis for discriminating participants with TBI from healthy controls. While dual-task and complex task gait were not assessed within the same study protocol, there were similar outcomes when walking with these additional tasks (i.e., slower gait in TBI groups), which may indicate that adding any additional task could highlight impairments. However, despite the reviewed studies finding gait differences in TBI with the increased cognitive (or cortical) demand of dual-tasks and complex tasks, the benefit over using single-task gait (a simpler and quicker task) remains unknown, as gait deficits were detected across the TBI spectrum (mTBI, modTBI, SevTBI) using single-task. This is further complicated by the lack of consistency in the type of dual-task, and the set-up of the complex task (obstacle crossing) makes it difficult to directly compare outcomes across studies, and therefore difficult to make any clinical assessment recommendations. Future studies should consider whether their protocols require increased task complexity in order to detect gait deficits, as performance of a single-task walk may be sufficient to detect deficits when comprehensively investigating gait with data-driven digital technologies.

### 4.4. Outcome Interpretation

While the reviewed studies found differences in gait in those with TBI compared to controls, or within TBI when examined using tasks of increasing complexity, there were substantive methodological limitations that impact the interpretation of the reported outcomes. Specifically, none of the reviewed studies examined gait impairment differences between the various severity levels of the injury (mTBI, modTBI, sevTBI), with few studies examining modTBI. This is likely a result of the difficulties in defining the various levels of TBI, as there were variations in the reported diagnostic criteria (i.e., some acute diagnosis was 7 days, others only hours, and chronic ranged from months to years post-injury) and the specific individuals involved in the diagnostics within the reviewed studies (i.e., athletic trainer or a team physician, or merely medical recorded screen). Without being able to clearly define the severity and stage of TBI using a standardised criteria and then examine gait across these sub-groups, it is difficult to determine whether gait could be an effective biomarker for determining diagnosis, severity level, prognosis or monitoring of this neurological condition. Additionally, none of the reviewed studies included area under the curve or receiver operating characteristic curve analysis for specificity or sensitivity of gait characteristics in determining TBI gait differences with controls, which limits the interpretation of results (i.e., there may be differences but they may have low diagnostic value [53]. Therefore, future studies are needed to develop standard procedures for examining gait impairment in TBI, which will aid in the determination of gait as a marker of TBI.

## 5. Conclusions

Gait was shown to be impaired in TBI within the reviewed studies regardless of the severity or stage of the injury, but the specific impairments and the outcomes of clinical relevance are yet to be fully established across the spectrum of the condition. Further research is required to establish standardized methods for gait assessment in TBI, which will help to determine the gait deficits at each severity level of injury (mTBI, modTBI, sevTBI) in larger well-defined cohorts to establish findings.

## Data Availability

Not applicable.

## Supplementary Materials

The following are available online at https://www.mdpi.com/article/10.3390/s22041480/s1, Table S1: List of articles that did not meet inclusion criteria and reason for exclusion.
