# Supplementary material for: Gait Impairment in Traumatic Brain Injury: A Systematic Review
Source: Sensors (Basel). 2022 Feb 14;22(4):1480. doi: 10.3390/s22041480 (PMC8875145; doi:10.3390/s22041480)
Supplement: Supplementary file 1 [file sensors-22-01480-s001.zip › sensors-1495672-supplementary.pdf]

Supplementary Table S1: List of articles that did not meet inclusion criteria and reason for exclusion

Citation	Reason for exclusion
Buckley, T.A., Munkasy, B.A. and Evans, K.M., (2014). Dual Task Gait Testing Identifies Lingering Post Concussion Impairments: 2170 Board# 7 May 29, 5: 45 PM-6: 45 PM. *Medicine & Science in Sports & Exercise*, *46*(5S), p.584.	Conference extract.
Catena, R.D., Van Donkelaar, P. and Chou, L.S., (2007). Cognitive task effects on gait stability following concussion. *Experimental brain research*, *176*(1), pp.23-31.	Inclusion of u18's.
Dugan, E.L., Shilt, J.S., Masterson, C.M. and Ernest, K.M., 2021. The use of inertial measurement units to assess gait and postural control following concussion. *Gait & Posture*, *83*, pp.262-267.	Inclusion of u18's.
Geurts, A.C., Ribbers, G.M., Knoop, J.A. and van Limbeek, J., (1996). Identification of static and dynamic postural instability following traumatic brain injury. *Archives of physical medicine and rehabilitation*, *77*(7), pp.639-644.	No control participants.
Howell, D.R., Osternig, L.R. and Chou, L.S., (2015). Return to activity after concussion affects dual-task gait balance control recovery. *Medicine and science in sports and exercise*, *47*(4), pp.673-680.	Inclusion of u18's.
Howell, D.R., Osternig, L.R., Christie, A.D. and Chou, L.S., (2016). Return to physical activity timing and dual-task gait stability are associated 2 months following concussion. *The Journal of head trauma rehabilitation*, *31*(4), pp.262-268.	Inclusion of u18's.
Howell, D.R., Oldham, J.R., DiFabio, M., Vallabhajosula, S., Hall, E.E., Ketcham, C.J., Meehan, W.P. and Buckley, T.A., (2017). Single-task and dual-task gait among collegiate athletes of different sport classifications: implications for concussion management. *Journal of applied biomechanics*, *33*(1), pp.24-31.	Prior TBI history.
Howell, D.R., Brilliant, A., Berkstresser, B., Wang, F., Fraser, J. and Meehan III, W.P., (2017). The association between dual-task gait after concussion and prolonged symptom duration. *Journal of neurotrauma*, *34*(23), pp.3288-3294.	Inclusion of u18's.
Howell, D.R., Stracciolini, A., Geminiani, E. and Meehan III, W.P., (2017). Dual-task gait differences in female and male adolescents following sport-related concussion. *Gait & posture*, *54*, pp.284-289.	Inclusion of u18's.
Howell, D.R., Osternig, L.R. and Chou, L.S., (2017). Single-task and dual-task tandem gait test performance after concussion. *Journal of science and medicine in sport*, *20*(7), pp.622-626.	Inclusion of u18's.
Howell, D.R., Buckley, T.A., Lynall, R.C. and Meehan III, W.P., (2018). Worsening dual-task gait costs after concussion and their association with subsequent sport-related injury. *Journal of neurotrauma*, *35*(14), pp.1630-1636.	Inclusion of u18's.
Howell, D.R., Osternig, L.R. and Chou, L.S., (2018). Detection of acute and long-term effects of concussion: Dual-task gait balance control versus computerized neurocognitive test. *Archives of physical medicine and rehabilitation*, *99*(7), pp.1318-1324.	Inclusion of u18's.
Howell, D.R., Stillman, A., Buckley, T.A., Berkstresser, B., Wang, F. and Meehan III, W.P., (2018). The utility of instrumented dual-task gait and tablet-based neurocognitive measurements after concussion. *Journal of science and medicine in sport*, *21*(4), pp.358-362.	Prior TBI history.
Howell, D.R., Bonnette, S., Diekfuss, J.A., Grooms, D.R., Myer, G.D., Wilson, J.C. and Meehan, W.P., (2020). Dual-task gait stability after concussion and subsequent injury: an exploratory investigation. *Sensors*, *20*(21), p.6297.	Inclusion of u18's.
Howell DR, Oldham J, Lanois C, Koerte I, Lin AP, Berkstresser B, et al. Dual-Task Gait Recovery after Concussion among Female and Male Collegiate Athletes. Med Sci Sports Exerc [Internet]. 2020 May 1 [cited 2021 Nov 9];52(5):1015. Available from:/pmc/articles/PMC7166163/	No control participants.

Leland, A., Tavakol, K., Scholten, J., Mathis, D., Maron, D. and Bakhshi, S., (2017). The role of dual tasking in the assessment of gait, cognition and community reintegration of veterans with mild traumatic brain injury. *Materia socio-medica*, *29*(4), p.251.	No control participants.
McCulloch, K.L., Buxton, E., Hackney, J. and Lowers, S., (2010). Balance, attention, and dual-task performance during walking after brain injury: associations with falls history. *The Journal of head trauma rehabilitation*, *25*(3), pp.155-163.	No control participants.
Niechwiej-Szwedo, E., Inness, E.L., Howe, J.A., Jaglal, S., McIlroy, W.E. and Verrier, M.C., (2007). Changes in gait variability during different challenges to mobility in patients with traumatic brain injury. *Gait & posture*, *25*(1), pp.70-77.	TBI severity and type not distinguished.
Williams, G., Morris, M.E., Schache, A. and McCrory, P.R., 2009. Incidence of gait abnormalities after traumatic brain injury. *Archives of physical medicine and rehabilitation*, *90*(4), pp.587-593.	Inclusion of u18's.
